# Lurking Under the Surface: Dercum’s Disease

**DOI:** 10.7759/cureus.17649

**Published:** 2021-09-01

**Authors:** Anthony Lam, William Aukerman, Bradley Winegarden, Shawna Morrissey

**Affiliations:** 1 General Surgery, Conemaugh Memorial Medical Center, Johnstown, USA; 2 Trauma and Acute Care Surgery, Conemaugh Memorial Medical Center, Johnstown, USA

**Keywords:** adipose, soft tissue mass, fat, adiposis dolorosa, dercum’s disease, painful subcutaneous masses, hyperechoic, orphan disease, lipoma, chronic pain

## Abstract

Adiposis dolorosa, also known as Dercum’s disease, is a rare disorder characterized by debilitating painful lipomas throughout the body. The prevalence and etiology of Dercum’s disease are unknown as mentioned in the National Organization of Rare Disorders. We present an interesting case of Dercum’s disease in a 53-year-old female who initially presented with a six-week history of painful subcutaneous masses. Ultrasound findings were suggestive of lipomas, however, her symptoms were debilitating beyond that of benign lipomas. She then represented with a rapidly increasing number of soft tissue masses manifesting throughout her body, as well as significant diffuse pain concentrating around these lesions within a short period of time following her initial presentation. The patient underwent surgical excision of a select number of these masses, with histopathology consistent with lipomas. Most cases of Dercum's disease are sporadic, and no guidelines exist regarding the treatment of the disease. Due to the rarity of this condition, in conjunction with simple lipomas typically presenting as painless masses, many patients may be misdiagnosed and neglected due to being falsely labeled as pain seeking or having their symptoms attributed to psychological disorders. Management, therefore, is complex and currently consists of a multidisciplinary approach employing multimodal treatments, including pain control, surgical excision, and psychotherapy. Although this condition has been described in the literature for over 100 years, there have been minimal advancements towards alleviating the suffering of these patients. We aimed to unearth and bring to light the reality and the suffering experienced by patients with Decrum's disease.

## Introduction

Adiposis dolorosa, also known as Dercum’s disease, is a fairly rare and understudied disease typically characterized by painful subcutaneous adipose tissue growth of varying size and localization throughout the body under the surface of the skin [1]. Due to the unacquainted nature of the condition, patients can be dismissed and belittled regarding the complexity of their complaints in proportion to the manifestation of physical signs. These abnormal tissue growths physically appear to be simple lipomas but unlike normal lipomas, the adipose tissue in Dercum’s disease bears significant pain and tenderness. Thus, the diagnosis of this condition is often dismissed with skepticism for drug-seeking behavior [2]. The definition of Dercum’s disease is, at minimum, proposed by Hansson et al. as overweight or obese patients with associated painful adipose tissue.

## Case presentation

We present a case of a 53-year-old female who presented with a six-week history of painful subcutaneous masses. Various soft tissue nodules had been present for multiple years; however, during the last few weeks prior to the initial evaluation, she developed an alarming increased number of widespread soft-tissue masses with significant tenderness in comparison. Her daily activities are often interrupted due to the menacing symptoms, causing significant attrition to her quality of life. Multiple attempts of over-the-counter trials of pain medications have failed to provide the patent with any relief and prescription pain medications are but a bandage for her illness. The patient has a significant past medical history including obesity (BMI 35), chronic atrial fibrillation, chronic obstructive pulmonary disease (COPD), diabetes, hypertension, and sick sinus syndrome status post atrial pacemaker placement.

During the initial presentation, ultrasound examination revealed hyperechoic foci in the subcutaneous tissues that were circumscribed without any Doppler flow, consistent with lipomas. The patient then underwent excision of multiple back nodules to further characterize the lesions, revealing a gross pathology report of adipose tissue with nodularity. An interesting point to note is that the rate at which new palpable nodules developed was intriguing. She developed four new identifiable lesions in various parts of the body in the short interval between her office visit and the date of the operation. Histopathology reports one of four specimens demonstrating portions of mature adipose tissue consistent with lipoma, and three of four specimens with mature adipocytes and branching network of small vessels consistent with angiolipoma.

She was seen in the postoperative period with a continued manifestation of these subcutaneous nodules throughout her body. The pain along the sites of excised lesions had improved but she continues to have significant tenderness along the areas of new masses. The patient's case was further reviewed and labeled with the diagnosis of Dercum’s disease - type 2 [3]. The patient again was seen in the surgery clinic at our institution with subsequent referral to endocrinology for further evaluation and to the pain clinic for multimodality treatment. In the interim, she continues to develop new painful collections of fatty tissue masses.

## Discussion

Epidemiology

The condition was first described by Francis Zavier Dercum, an American neurologist, in various articles of writings in 1888 and 1892, with the terminology of adiposis dolorosa. It is estimated for approximately 140-160 total publications regarding the illness, with a majority of them were case reports. Dercum’s disease is classified as an orphan disease within the National Organization for Rare Disorders (NORD) and Orphanet (ORPHA:36397). The disease is found to be 5-10x more common in females, generally after menopause, between the ages of 35 years and 50 years [4]. Due to the scarcity of data, there are no adequate studies calculating the prevalence or incidence of the disease.

Laboratory/pathology

**Figure 1:**
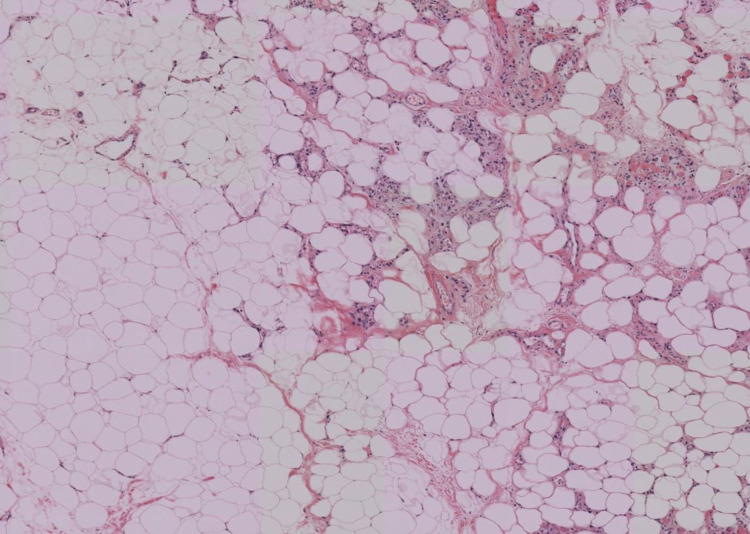
Low-power image of an excised painful nodule (specimen A) with infiltrates of inflammatory cells. Hematoxylin-eosin staining. 4× objective

**Figure 2:**
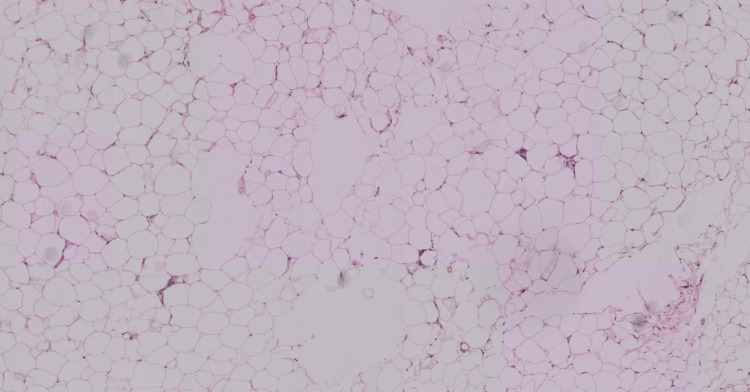
Low-power image of an excised painful nodule (specimen B) from the back. Hematoxylin-eosin staining. 4× objective

No biomarkers associated with Dercum’s have been identified. Histopathology analysis of tissue biopsies are consistent with fatty connective tissues and are virtually indistinguishable from lipomas, as seen on our patient's histology in Figures 1, 2 [2].

Imaging

**Figure 3:**
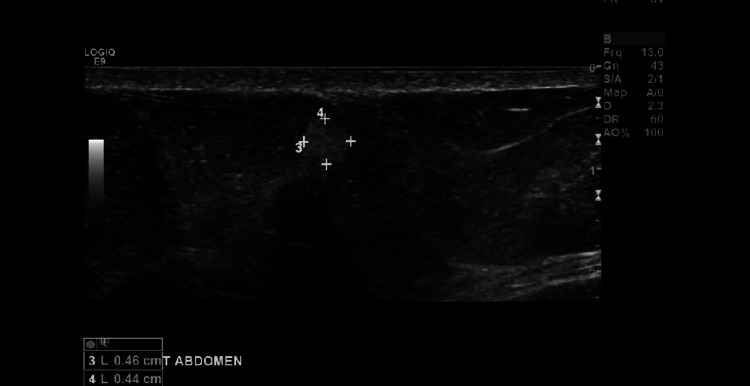
Ultrasound image of a 0.46 x 0.44 x 0.35 cm right abdominal soft tissue mass

**Figure 4:**
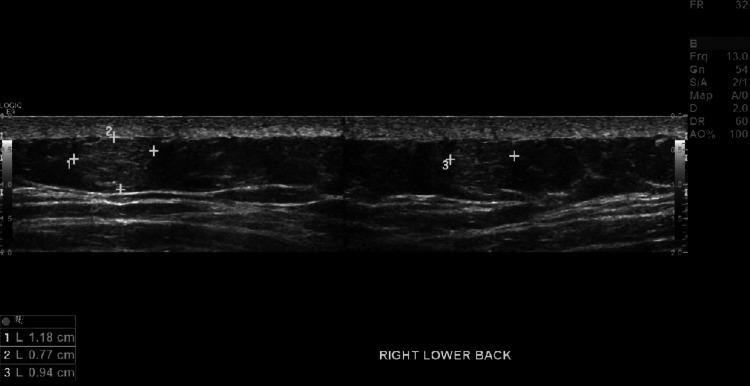
Ultrasound image of a 1.18 x 0.94 x 0.77 cm right lower back soft tissue mass

**Figure 5:**
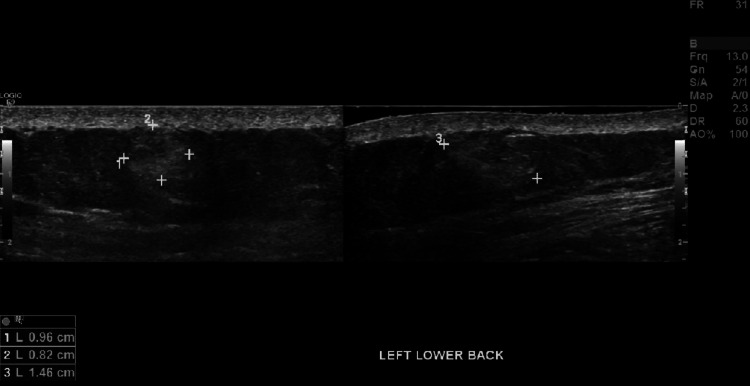
Ultrasound image of a 1.46 x 0.96 x 0.82 cm left lower back soft tissue mass

**Figure 6:**
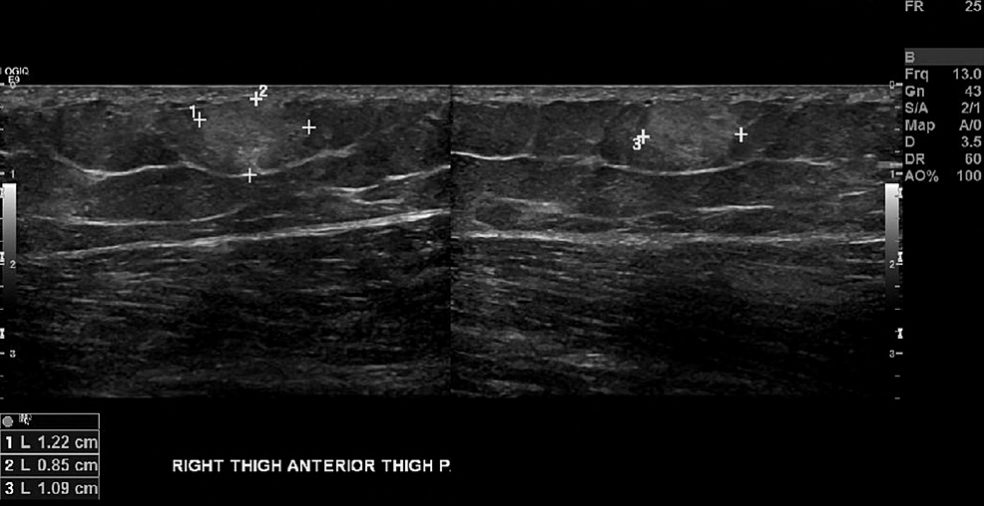
Ultrasound image of a 1.22 x 1.09 x 0.85 cm right anterior thigh soft tissue mass

**Figure 7:**
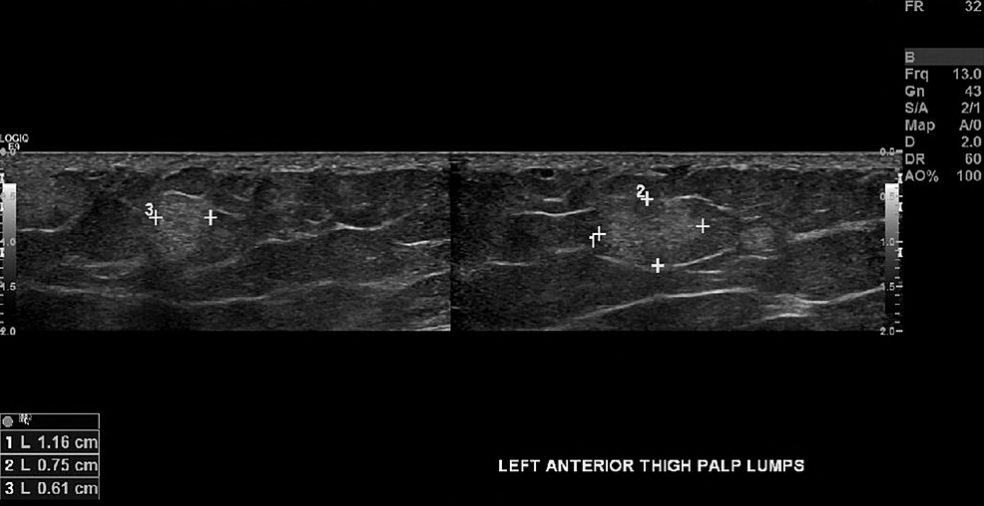
Ultrasound image of a 1.16 x 0.61 x 0.75 cm left anterior thigh soft tissue mass

Evaluation of soft tissue masses typically consists of CT, MRI, or ultrasound (U/S) to further characterize lesions. Ultrasound studies are cost-effective modalities without the adverse radiation associated with CT scans, yet provide excellent sensitivities for evaluation of superficial soft tissue masses. The majority of lesions associated with Dercum’s disease were < 2 cm with a long axis parallel to the skin, and on ultrasound, the lesions were hyperechoic without demonstrable flow on Doppler imaging, consistent with our patient’s ultrasound findings as seen in Figures 3-7 [5].

Diagnosis

Based upon a complete history and thorough physical examination, Dercum’s disease is typically characterized by two main diagnostic criteria: (1) obesity and (2) chronic pain associated with hyperplastic/hypertrophic adipose tissue. The diagnosis of Dercum’s disease is one of exclusion of other subcutaneous soft tissue conditions [2]. Four different variants of Dercum’s disease have been proposed by Hasson et al. as outlined in Table 1 [1].

**Table 1:** Classification of Dercum’s disease This table is adapted from Hansson et al. [1].

Type	Description
Type 1: Generalized diffuse form	Widespread painful adipose tissue without lipoma
Type 2: Generalized nodular form	Widespread painful adipose tissue and increased sensitivity surrounding lipomas
Type 3: Localized nodular form	Pain strictly surrounding lipomas
Type 4: Juxta-articular form	Painful adipose tissue near large joints

## Conclusions

This case report brings further attention to a rare disorder affecting more patients than previously thought. The broad clinical picture surrounding a common benign finding, such as a lipoma, makes the differential more difficult. When adding patient factors to the decision-making process, clinicians may be less likely to diagnose Dercum’s disease. However, there has yet to be one definitive method of diagnosing, treating, and/or preventing this disease, therefore, more research is needed. As the world's population and obesity rates continue to grow, Dercum’s disease should be another differential that clinicians can keep in their armamentarium.
